# Relationship of Smokefree Laws and Alcohol Use with Light and Intermittent Smoking and Quit Attempts among US Adults and Alcohol Users

**DOI:** 10.1371/journal.pone.0137023

**Published:** 2015-10-07

**Authors:** Nan Jiang, MariaElena Gonzalez, Pamela M. Ling, Stanton A. Glantz

**Affiliations:** 1 Center for Tobacco Control Research and Education, University of California San Francisco, San Francisco, California, United States of America; 2 School of Public Health, The University of Hong Kong, Hong Kong, China; 3 School of Social Sciences, Humanities & Arts, University of California Merced, Merced, California, United States of America; University of Florida, UNITED STATES

## Abstract

**Introduction:**

Light and intermittent smoking (LITS) has become increasingly common. Alcohol drinkers are more likely to smoke. We examined the association of smokefree law and bar law coverage and alcohol use with current smoking, LITS, and smoking quit attempts among US adults and alcohol drinkers.

**Methods:**

Cross-sectional analyses among a population-based sample of US adults (n = 27,731) using restricted data from 2009 National Health Interview Survey and 2009 American Nonsmokers' Rights Foundation United States Tobacco Control Database. Multivariate logistic regression models examined the relationship of smokefree law coverage and drinking frequency (1) with current smoking among all adults; (2) with 4 LITS patterns among current smokers; and (3) with smoking quit attempts among 6 smoking subgroups. Same multivariate analyses were conducted but substituted smokefree bar law coverage for smokefree law coverage to investigate the association between smokefree bar laws and the outcomes. Finally we ran the above analyses among alcohol drinkers (n = 16,961) to examine the relationship of smokefree law (and bar law) coverage and binge drinking with the outcomes. All models controlled for demographics and average cigarette price per pack. The interactions of smokefree law (and bar law) coverage and drinking status was examined.

**Results:**

Stronger smokefree law (and bar law) coverage was associated with lower odds of current smoking among all adults and among drinkers, and had the same effect across all drinking and binge drinking subgroups. Increased drinking frequency and binge drinking were related to higher odds of current smoking. Smokefree law (and bar law) coverage and drinking status were not associated with any LITS measures or smoking quit attempts.

**Conclusions:**

Stronger smokefree laws and bar laws are associated with lower smoking rates across all drinking subgroups, which provides further support for these policies. More strict tobacco control measures might help reduce cigarette consumption and increase quit attempts.

## Introduction

Light and intermittent smoking (LITS) including nondaily smoking, light and very light smoking has become increasingly common [1–3]. Earlier work has found that stronger smokefree law coverage is associated with lower smoking prevalence and cigarette consumption [4,5], but it is not known if stronger smokefree law coverage is related to increased LITS. Bars and nightclubs are key public venues where drinkers smoke. However, no study has investigated whether stronger smokefree bar law coverage is associated with lower smoking prevalence and increased LITS.

Smokefree laws are associated with increased quit attempts. Smokefree workplaces increased the odds of quit attempts and cessation [6,7], and strong local restaurant laws were related to increased quit attempts [8]. However, it remains unknown if smokefree law coverage and bar law coverage are related to quit attempts in different types of smokers, especially those light and intermittent smokers.

Alcohol drinkers are more likely to smoke and are disproportionally suffered from tobacco-related diseases [9–19]. It is unknown how smokefree law (and bar law) coverage affects current smoking, LITS, and smoking quit attempts among drinkers. Would the relationship, if any, varies by drinking frequency or binge drinking has not been studied. The association between smoking and drinking became stronger with the heavier use of either substance [10,13,14], and the high frequency of alcohol consumption and alcohol dependence make it harder to quit smoking [20,21]. However, no study has examined whether alcohol use and binge drinking are associated with less LITS among current drinking smokers. No study has investigated the relationship between alcohol use and smoking quit attempts among drinkers who reported different smoking patterns.

In addition to the above gaps in knowledge, prior research on smokefree law coverages only assessed state laws [5,22] despite the fact that local laws are often stronger than the state laws. Or the study classified the law coverage into dichotomous groups (strong vs. others) [4] without accounting for laws with different combinations of venue coverage and the percentage of county population covered.

This study fills these gaps. It examines the relationship of smokefree law coverage, smokefree bar law coverage, and alcohol use (or binge drinking) with (1) current (past 30-day) smoking among US adults in general and among alcohol drinkers, (2) 4 LITS categories (i.e., nondaily, very light daily, very light nondaily, and infrequent smoking) among current smokers and among current drinking smokers, and (3) smoking quit attempts among 6 types of smokers (i.e., current, daily, nondaily, very light daily, very light nondaily, and infrequent smokers) and among drinkers who reported different smoking patterns. We used smokefree law coverage score [23] that accounts for the venues (i.e., public workplaces, private workplaces, restaurants, and bars) and the percentage of county population covered by the laws. We found that stronger smokefree law (and bar law) coverage was associated with lower odds of current smoking across all drinking and binge drinking subgroups, but the law coverage and alcohol use were not associated with any LITS status or smoking quit attempts.

## Methods

We linked the restricted data from 2009 National Health Interview Survey (NHIS), a cross-sectional multi-stage probability sample of the noninstitutionalized civilian US population, to smokefree law coverage data from 2009 American Nonsmokers' Rights Foundation (ANRF) United States Tobacco Control Database [24]. Our sample of adults aged ≥18 years who completed the adult core questionnaires included 27,731 respondents (response rate 65.4%) [25]. This study did not require the review of institutional review board because it is a secondary data analysis of de-identified data from the NHIS.

### Dependent variables

**Fig 1:**
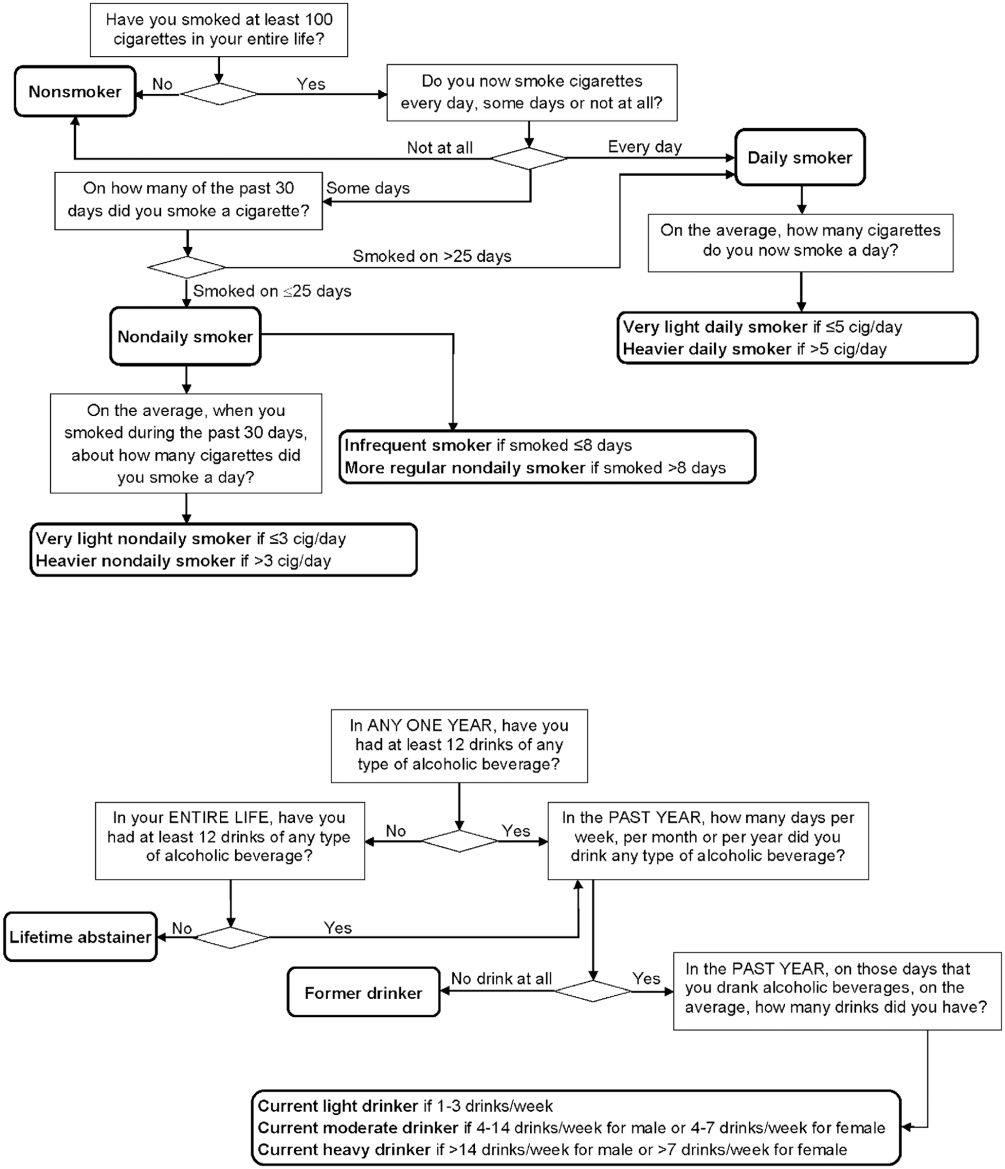
Classification of respondents. (a) Classification of smoking status. (b) Classification of alcohol drinking status.

Fig 1a shows the smoking subgroups. *Current smokers* smoked at least 100 cigarettes in lifetime and currently smoked “every day” or “some days”, and were dichotomized into *nondaily* and *daily smokers*. Nondaily smokers smoked “some days” and on ≤25 days in past 30-day [26–28]; daily smokers smoked “every day”, or “some days” but on >25 days in past 30-day. Daily smokers were dichotomized into *very light* (≤5 cig/day) and *heavier daily smokers* (>5 cig/day) following earlier research [29–32].

Because low consumption smoking for nondaily smokers can be conceptualized as either smoking fewer days per month or fewer cig/day, we tested two definitions of low consumption nondaily smoking: *very light nondaily smoker* and *infrequent smoker*. Nondaily smokers were classified as very light (≤3 cig/day) or heavier nondaily smokers (>3 cig/day). We used 3 cig/day cutoff for nondaily smokers but 5 cig/day for daily smokers because a prior study of very light smokers (≤5 cig/day) found that very light nondaily smokers smoked fewer cig/day than very light daily smokers [29]. Infrequent smokers smoked on ≤8 days in past 30-day, and more regular nondaily smokers smoked on 9–25 days. The 8-day cutoff was used because occasional smokers reported smoking a median of 8 days/month in a prior study [33].

*Quit attempts* was measured among current smokers by the question, “During the past 12 months, have you stopped smoking for more than one day because you were trying to quit smoking?”

### Independent variables

We used respondent’s county to determine smokefree law coverage score and his/her state for cigarette pack price, and linked these variables to NHIS data using Federal Information Processing Standards (FIPS) codes.

We used ANRF United States Tobacco Control Database and the Census Estimated Population to calculate the *smokefree law coverage score* [23] in 2009 for each county. The smokefree law coverage score is a continuous variable, ranging from 0 to 1, with larger values representing more smokefree venues and a larger proportion of county population covered by the law. While smokefree law coverage strength may vary across venues, laws covering several venues are often passed simultaneously [34]. The simultaneous passage of smokefree laws as well as their overlapping nature in population coverage introduces collinearity when entering them separately into a model. Therefore, it is necessary to calculate a smokefree law coverage score to capture both the dimension and breadth of smokefree law coverage within a county. We computed the fraction of county population covered by 100% smokefree public and private workplaces, restaurants, or bars to capture the breadth of law coverage then averaged the four results to account for the venues and percentage of population covered by 100% smokefree laws in a county, accounting for sub-state and sub-county variation in coverage [23]. To investigate the specific effects of smokefree bar law coverage, we also calculated a similar *smokefree bar law coverage score*.

Following the report *Health*, *United States*, *2009* [25,35], respondents were classified into 5 drinking subgroups (Fig 1b): “lifetime abstainer” (<12 drinks in lifetime); “former drinker” (≥12 lifetime drinks but none in the past year); “current light drinker” (≥12 lifetime drinks and 1–3 drinks/week in the past year); “current moderate drinker” (4–14 drinks/week for males; 4–7 drinks/week for females); and “current heavy drinker” (≥14 drinks/week for males; ≥7 drinks/week for females). *Binge drinking* was measured among current drinkers with the question “In the past year, on how many days did you have 5 or more drinks of any alcoholic beverage?” Respondents were dichotomously categorized into binge (≥5 drinks on at least one day in the past 12 months) and non-binge drinkers.

*Demographics* included gender, age group (18–20, 21–24, 25–44, 45–64, and 65 and older), race/ethnicity (non-Hispanic White, non-Hispanic Black, non-Hispanic Asian/Pacific Islander (API) and other, and Hispanic), education (less than high school, high school graduate or general equivalency diploma (GED), some college, and college graduate), and poverty status (poor, near poor, not poor, and unreported [36]). We used the Centers for Disease Control and Prevention’s (CDC) report on age groups [37], but divided the 18-24-year-olds into two groups because smokefree bar laws apply directly to adults aged 21 years and older. *Average cigarette price per package* was from *The Tax Burden on Tobacco* [38] adjusted for the timing of changes in state and federal taxes and underlying industry prices [39].

### Statistical analysis

Data analysis was conducted in December 2013. The actual computer runs were conducted by the CDC Research Data Center (RDC) using SAS and Stata code submitted through RDC’s remote access system. The analysis accounted for complex sample design and weighted respondents to reflect the probability of selection and adjustments for non-response and post-stratification [25]. All variance inflation factors were between 1.02 and 1.15, indicating that multicollinearity was not an issue.

First, we used multivariate logistic regression to determine if smokefree law coverage and alcohol consumption frequency were associated with current smoking among all adults. The model included the interaction of smokefree law coverage score and drinking frequency, and controlled for demographics and average cigarette pack price. We then substituted the smokefree bar law coverage score for smokefree law coverage in the model to examine the association between bar law coverage and current smoking among all adults. Next, we conducted the above multivariate analyses and limited to alcohol drinkers (n = 16,961) to assess the relationship of smokefree law (and bar law) coverage and binge drinking status with current smoking.

Second, we conducted separate multivariate logistic regression models to assess if smokefree law coverage and alcohol use frequency were associated with LITS patterns. Specifically, we examined the relationship of smokefree law coverage and drinking frequency with (1) nondaily smoking among current smokers, (2) very light daily smoking among daily smokers, (3) very light nondaily smoking among nondaily smokers, and (4) infrequent smoking among nondaily smokers. The interactions of smokefree law coverage score and drinking frequency were included in all models which also controlled for demographics and cigarette price. We then ran the same models but using smokefree bar law coverage score to assess the relationship between smokefree bar law coverage and each of the 4 LITS patterns. Next, we limited our analysis to alcohol drinkers to examine the association between smokefree law (and bar law) coverage and binge drinking and LITS.

Finally, we conducted separate multivariate logistic regression models within 6 smoking subgroups (i.e., current smokers, daily smokers, nondaily smokers, very light daily smokers, very light nondaily smokers, and infrequent smokers) to examine if smokefree law coverage and drinking frequency were associated with smoking quit attempts. Models included interactions of smokefree law coverage score and drinking frequency controlling for demographics and cigarette price. We then substituted smokefree bar law coverage for smokefree law coverage to examine the relationship between smokefree bar law coverage and smoking quit attempts. The same analyses were conducted among alcohol drinkers who reported each of the 6 smoking patterns to assess the relationship between smokefree law (and bar law) coverage and binge drinking on smoking quit attempts.

## Results

S1 Table depicts the sample characteristics. The mean 100% smokefree law coverage score was 0.63 and 0.52 for bar law coverage. The average cigarette price was $4.76 per pack. 20.6% (95% confidence interval: 19.9, 21.3) of adults reported current smoking (Table 1). Among drinkers, current smoking rate increased with drinking frequency. Binge drinkers' smoking prevalence was twice as high as that of non-binge drinkers. 65.1% (64.3, 65.9) of adults reported current (past 12-month) drinking, and 35.6% (34.4, 36.8) of drinkers reported binge drinking (data not shown in tables). Nearly half of current smokers reported smoking quit attempts in the past 12 months. Alcohol abstainers and light drinkers reported higher smoking quit attempt rates than heavy drinkers.

**Table 1:** Prevalence of current smoking, light and intermittent smoking status, and quit attempts. *Note*. CI = confidence interval.

	Current smoker^a^		Daily smoker^b^		Nondaily smoker^c^		Very light daily smoker^d^		Very light nondaily smoker^e^		Infrequent smoker^f^		Quit attempt^g^ among current smoker	
	n	% [95% CI]	n	% [95% CI]	n	% [95% CI]	n	% [95% CI]	n	% [95% CI]	n	% [95% CI]	n	% [95% CI]
**Total**	**5578**	**20.6 [19.9, 21.3]**	**4416**	**16.6 [15.9, 17.3]**	**1125**	**3.9 [3.6, 4.2]**	**718**	**2.3 [2.1, 2.6]**	**661**	**2.3 [2.0, 2.5]**	**422**	**1.4 [1.2, 1.6]**	**2632**	**46.8 [44.9, 48.7]**
**Age group (years)**														
18–20	189	19.0 [15.7, 22.3]	125	13.2 [10.3, 16.1]	57	5.1 [3.5, 6.7]	36	3.5 [2.1, 5.0]	42	3.7 [2.4, 5.0]	15	1.0 [0.4, 1.6]	95	50.6 [41.8, 59.4]
21–24	401	23.7 [20.8, 26.6]	275	17.7 [14.9, 20.5]	123	5.8 [4.6, 7.1]	79	3.8 [2.7, 4.9]	82	4.0 [2.9, 5.0]	45	2.2 [1.4, 2.9]	210	56.0 [49.0, 62.9]
25–44	2304	24.0 [22.8, 25.1]	1768	18.7 [17.7, 19.8]	525	5.1 [4.6, 5.7]	278	2.4 [2.1, 2.8]	321	3.2 [2.7, 3.6]	207	2.0 [1.7, 2.3]	1138	48.3 [45.7, 50.8]
45–64	2146	21.9 [20.7, 23.2]	1798	18.8 [17.6, 20.0]	340	3.1 [2.7, 3.5]	250	2.3 [1.9, 2.7]	177	1.6 [1.3, 1.9]	123	1.1 [0.8, 1.3]	973	43.9 [41.0, 46.9]
65 and above	538	9.5 [8.5, 10.5]	450	7.9 [7.0, 8.8]	80	1.5 [1.1, 2.0]	75	1.1 [0.8, 1.4]	39	0.6 [0.4, 0.9]	32	0.7 [0.4, 1.0]	216	40.0 [34.5, 45.4]
**Gender**														
Male	2828	23.5 [22.4, 24.5]	2186	18.5 [17.5, 19.5]	622	4.8 [4.3, 5.3]	322	2.3 [2.0, 2.6]	359	2.7 [2.4, 3.1]	244	1.8 [1.5, 2.0]	1299	46.0 [43.5, 48.5]
Female	2750	17.9 [17.1, 18.7]	2230	14.8 [14.1, 15.5]	503	3.0 [2.7, 3.4]	396	2.4 [2.0, 2.7]	302	1.8 [1.6, 2.1]	178	1.1 [0.9, 1.3]	1333	47.8 [45.1, 50.4]
**Race/ethnicity**														
White, non-Hispanic	3554	22.2 [21.3, 23.1]	2942	18.4 [17.6, 19.3]	593	3.6 [3.3, 4.0]	286	1.7 [1.5, 2.0]	328	2.0 [1.7, 2.3]	208	1.3 [1.1, 1.5]	1595	45.0 [42.9, 47.2]
Black, non-Hispanic	968	21.3 [19.7, 22.9]	760	16.7 [15.2, 18.2]	200	4.4 [3.6, 5.2]	190	4.2 [3.4, 4.9]	113	2.5 [1.9, 3.1]	54	1.2 [0.8, 1.5]	520	52.0 [47.5, 56.4]
API and others, non-Hispanic	272	14.5 [12.4, 16.5]	211	11.0 [9.2, 12.8]	58	3.4 [2.3, 4.5]	53	2.8 [1.9, 3.6]	34	1.9 [1.1, 2.7]	24	1.2 [0.6, 1.8]	132	51.3 [43.2, 59.4]
Hispanic	784	14.5 [13.2, 15.9]	503	9.5 [8.4, 10.6]	274	4.9 [4.1, 5.8]	189	3.7 [3.0, 4.4]	186	3.5 [2.8, 4.3]	136	2.3 [1.8, 2.8]	385	51.9 [46.2, 57.6]
**Education**														
0–12 years (no diploma)	1154	27.4 [25.5, 29.2]	943	23.4 [21.5, 25.2]	202	3.9 [3.2, 4.5]	181	3.5 [2.8, 4.2]	130	2.5 [1.9, 3.0]	88	1.5 [1.1, 1.9]	527	42.6 [38.7, 46.4]
High school graduate/GED	1963	27.7 [26.3, 29.1]	1636	23.5 [22.2, 24.7]	315	4.1 [3.5, 4.7]	232	3.0 [2.5, 3.5]	172	2.1 [1.7, 2.6]	103	1.2 [0.8, 1.5]	915	45.4 [42.4, 48.5]
Some college (no degree) or associate degree	1736	20.8 [19.6, 22.0]	1368	16.5 [15.3, 17.6]	358	4.2 [3.7, 4.8]	205	2.2 [1.8, 2.6]	217	2.7 [2.2, 3.1]	125	1.5 [1.1, 1.8]	856	50.8 [47.6, 54.0]
Undergraduate/graduate degree	698	9.3 [8.4, 10.1]	451	6.0 [5.3, 6.7]	242	3.2 [2.7, 3.7]	96	1.1 [0.9, 1.4]	137	1.9 [1.5, 2.2]	101	1.5 [1.1, 1.9]	323	46.9 [42.5, 51.3]
**Poverty status** ^**h**^														
<100% (Poor)	1256	31.1 [29.1, 33.0]	1023	25.8 [24.0, 27.6]	225	5.1 [4.3, 6.0]	203	4.5 [3.8, 5.3]	125	2.6 [2.1, 3.2]	79	1.7 [1.2, 2.1]	610	46.6 [42.6, 50.5]
100–199% (Near poor)	1123	25.2 [23.5, 27.0]	895	21.0 [19.3, 22.6]	224	4.2 [3.5, 4.9]	150	3.0 [2.4, 3.6]	149	2.9 [2.3, 3.5]	78	1.4 [1.0, 1.8]	539	47.4 [43.9, 50.9]
≥200% (Not poor)	2698	18.1 [17.3, 18.9]	2099	14.3 [13.5, 15.1]	586	3.7 [3.4, 4.1]	291	1.8 [1.5, 2.0]	341	2.2 [1.9, 2.5]	222	1.4 [1.1, 1.6]	1264	46.8 [44.4, 49.2]
Unspecified	501	17.0 [15.1, 18.9]	399	13.6 [11.9, 15.3]	90	3.0 [2.3, 3.8]	74	2.2 [1.6, 2.7]	46	1.5 [0.9, 2.1]	43	1.4 [0.9, 1.9]	219	46.2 [39.9, 52.4]
**Drinking status** ^**i**^														
Lifetime abstainer	493	8.1 [7.1, 9.1]	394	6.7 [5.7, 7.6]	90	1.3 [1.0, 1.7]	105	1.5 [1.1, 1.9]	57	0.9 [0.6, 1.2]	35	0.5 [0.3, 0.6]	242	50.0 [44.6, 55.3]
Former drinker	934	22.9 [21.1, 24.7]	825	20.4 [18.7, 22.2]	100	2.3 [1.8, 2.9]	121	2.7 [2.1, 3.4]	49	1.2 [0.7, 1.6]	35	0.8 [0.4, 1.1]	418	45.1 [40.9, 49.4]
Current light drinker	2349	20.2 [19.3, 21.2]	1862	16.3 [15.4, 17.3]	482	3.9 [3.4, 4.3]	303	2.2 [1.9, 2.5]	308	2.5 [2.1, 2.8]	184	1.4 [1.2, 1.6]	1183	49.9 [47.2, 52.5]
Current moderate drinker	1043	25.6 [23.8, 27.4]	740	18.4 [16.9, 19.9]	299	7.1 [6.1, 8.1]	121	2.5 [2.0, 3.1]	167	4.2 [3.4, 5.0]	108	2.7 [2.1, 3.3]	486	45.9 [42.0, 49.8]
Current heavy drinker	642	46.7 [43.6, 49.8]	504	38.0 [34.9, 41.0]	138	8.7 [6.9, 10.6]	59	4.5 [3.0, 6.0]	73	3.5 [2.6, 4.5]	53	3.3 [2.1, 4.4]	259	39.4 [34.4, 44.3]
**Binge drinking** ^**j**^ **(among current drinkers)**														
No	1986	17.4 [16.4, 18.4]	1581	14.0 [13.1, 14.9]	402	3.3 [2.9, 3.8]	264	2.0 [1.7, 2.3]	246	2.0 [1.7, 2.3]	146	1.2 [0.9, 1.4]	958	47.8 [44.9, 50.8]
Yes	1967	34.5 [32.9, 36.1]	1458	26.3 [24.8, 27.9]	503	8.1 [7.2, 9.0]	209	3.3 [2.7, 3.9]	293	4.7 [4.0, 5.4]	197	3.2 [2.6, 3.7]	943	47.1 [44.2, 50.1]
**Smokefree law coverage score**	0.59		0.58		0.61		0.59		0.62		0.62		0.59	
**Smokefree bar law coverage score**	0.47		0.46		0.52		0.49		0.54		0.54		0.49	
**Cigarette pack price (US dollar)**	4.72		4.71		4.75		4.78		4.79		4.79		4.76	

^a^Current smokers smoked at least 100 cigarettes in their lifetime and smoked “every day” or “some days” now.

^b^Daily smokers smoked “every day” now, or if they smoked “some days,” they smoked on >25 days in the past 30 days.

^c^Nondaily smokers smoked “some days” now and smoked on ≤25 days in the past 30 days.

^d^Very light daily smokers are daily smokers who smoked ≤5 cigarettes per day.

^e^Very light nondaily smokers are nondaily smokers who smoked ≤3 cigarettes per day.

^f^Infrequent smokers are nondaily smokers who smoked on ≤8 days in the past 30 days.

^g^Smoking respondent reported that he/she had stopped smoking for more than one day because he/she was trying to quit smoking in the past 12 months.

^h^Poverty status is a ratio of family income to the appropriate poverty threshold (given family size and number of children) defined by the US Census Bureau. “Poor” adults reported a family income below the poverty threshold. “Near poor” adults had a family income of 100–199% of the poverty threshold. “Not poor” adults reported a family income of 200% of the poverty threshold or greater.

^i^Lifetime abstainers had fewer than 12 drinks in lifetime; Former drinkers had at least 12 drinks in lifetime, but none in past year; Current light drinkers drank 1–3 drinks per week in past year; Current moderate drinkers drank 4–14 drinks per week for male and 4–7 drinks per week for female; Current heavy drinkers drank >14 drinks per week for male and >7 drinks per week for female.

^j^Binge drinkers drank ≥5 drinks on at least one day in the past 12 months.

### Smokefree law coverage, alcohol use, and current smoking or LITS

Among all adults, higher smokefree law coverage score was associated with lower current smoking (adjusted odds ratio [OR] = 0.80, *p* = .014, for score of 1 vs. 0), but not with any LITS patterns among smokers (Table 2). Drinking frequency was associated with current smoking: compared to light drinkers, lifetime abstainers (OR = 0.37, *p* < .001) had lower odds of being current smokers, moderate (OR = 1.68, *p* < .001) and heavy drinkers (OR = 3.94, *p* < .001) had higher odds of current smoking. Interactions of smokefree law coverage and drinking were not significant (*p* = .158-.845), indicating smokefree law coverage had similar relationship with smoking across all drinking subgroups.

**Table 2:** Relationship of smokefree law coverage and alcohol use with smoking. *Note*. AOR = adjusted odds ratio; CI = confidence interval.

Subpopulation	Adult	Current smoker^a^	Daily smoker^b^	Nondaily smoker^c^	
Outcome	Current smoker	Nondaily smoker	Very light daily smoker^d^	Very light nondaily smoker^e^	Infrequent smoker^f^
	AOR (95% CI)	AOR (95% CI)	AOR (95% CI)	AOR (95% CI)	AOR (95% CI)
N	27055	5411	4289	1047	1101
**Smokefree law coverage score**	0.80 (0.67, 0.96)*	0.86 (0.59, 1.24)	0.96 (0.60, 1.53)	0.93 (0.48, 1.79)	0.93 (0.48, 1.80)
**Drinking status** ^g^					
Lifetime abstainer	0.37 (0.29, 0.48)***	0.82 (0.43, 1.54)	1.26 (0.63, 2.51)	0.95 (0.27, 3.40)	0.99 (0.40, 2.43)
Former drinker	1.02 (0.81, 1.30)	0.49 (0.28, 0.83)**	0.95 (0.56, 1.60)	0.41 (0.15, 1.08)	1.11 (0.32, 3.79)
Current light drinker	1.00	1.00	1.00	1.00	1.00
Current moderate drinker	1.68 (1.34, 2.11)***	1.19 (0.80, 1.78)	0.87 (0.49, 1.56)	0.78 (0.36, 1.67)	0.69 (0.29, 1.60)
Current heavy drinker	3.94 (2.87, 5.41)***	0.63 (0.36, 1.10)	1.83 (0.80, 4.18)	0.26 (0.08, 0.81)*	1.35 (0.37, 4.92)
**Age group (years)**					
18–20	0.83 (0.64, 1.07)	3.14 (1.99, 4.98)***	3.39 (1.92, 6.00)***	2.63 (1.19, 5.82)*	0.43 (0.18, 1.01)
21–24	0.99 (0.82, 1.19)	2.00 (1.42, 2.82)***	2.09 (1.37, 3.18)***	2.44 (1.32, 4.53)**	1.08 (0.62, 1.89)
25–44	1.29 (1.16, 1.42)***	1.52 (1.24, 1.86)***	1.01 (0.79, 1.30)	1.25 (0.85, 1.84)	0.95 (0.65, 1.39)
45–64	1.00	1.00	1.00	1.00	1.00
65 and above	0.33 (0.28, 0.38)***	1.38 (0.97, 1.95)	1.28 (0.89, 1.84)	0.71 (0.34, 1.48)	1.29 (0.67, 2.48)
**Female**	0.86 (0.80, 0.94)***	0.95 (0.79, 1.15)	1.44 (1.12, 1.85)***	1.27 (0.91, 1.76)	0.97 (0.70, 1.35)
**Race/ethnicity**					
White, non-Hispanic	1.00	1.00	1.00	1.00	1.00
Black, non-Hispanic	0.77 (0.68, 0.87)***	1.63 (1.25, 2.11)***	3.55 (2.71, 4.67)***	1.35 (0.84, 2.17)	0.77 (0.47, 1.26)
API and others, non-Hispanic	0.88 (0.73, 1.07)	1.38 (0.90, 2.11)	3.39 (2.13, 5.39)***	0.94 (0.43, 2.05)	0.84 (0.42, 1.66)
Hispanic	0.35 (0.31, 0.41)***	2.83 (2.15, 3.71)***	7.04 (5.26, 9.42)***	2.94 (1.83, 4.74)***	1.70 (1.13, 2.58)*
**Education**					
0–12 years (no diploma)	6.12 (5.16, 7.26)***	0.26 (0.19, 0.37)***	0.47 (0.31, 0.70)***	1.07 (0.58, 1.97)	0.76 (0.42, 1.38)
High school graduate/GED	4.68 (4.09, 5.35)***	0.32 (0.24, 0.42)***	0.52 (0.36, 0.76)**	0.72 (0.43, 1.20)	0.48 (0.29, 0.81)**
Some college (no diploma)/associate degree	2.74 (2.43, 3.10)***	0.47 (0.36, 0.61)***	0.58 (0.40, 0.83)**	1.11 (0.70, 1.76)	0.64 (0.42, 0.99)*
Undergraduate/graduate degree	1.00	1.00	1.00	1.00	1.00
**Poverty status** ^h^					
<100% (Poor)	1.96 (1.73, 2.22)***	0.82 (0.65, 1.03)	1.10 (0.82, 1.48)	0.54 (0.35, 0.85)**	0.90 (0.54, 1.49)
100–199% (Near poor)	1.48 (1.31, 1.67)***	0.78 (0.62, 0.98)*	0.99 (0.74, 1.33)	1.20 (0.78, 1.85)	0.89 (0.59, 1.35)
≥200% (Not poor)	1.00	1.00	1.00	1.00	1.00
Unspecified	1.02 (0.89, 1.17)	0.89 (0.63, 1.26)	1.28 (0.90, 1.82)	0.76 (0.41, 1.43)	1.48 (0.83, 2.62)
**Cigarette pack price (US dollar)**	1.03 (0.97, 1.09)	1.01 (0.91, 1.12)	1.13 (0.98, 1.30)	1.15 (0.94, 1.42)	1.05 (0.85, 1.30)
**Smokefree law coverage × drinking status**	F_(4, 297)_ = 1.67; *p* = .158	F_(4, 297)_ = 1.18; *p* = .320	F_(4, 297)_ = 1.32; *p* = .261	F_(4, 281)_ = 0.35; *p* = .845	F_(4, 282)_ = 0.54; *p* = .705

^a^Current smokers smoked at least 100 cigarettes in their lifetime and smoked “every day” or “some days” now.

^b^Daily smokers smoked “every day” now, or if they smoked “some days,” they smoked on >25 days in the past 30 days.

^c^Nondaily smokers smoked “some days” now and smoked on ≤25 days in the past 30 days.

^d^Very light daily smokers are daily smokers who smoked ≤5 cigarettes per day.

^e^Very light nondaily smokers are nondaily smokers who smoked ≤3 cigarettes per day.

^f^Infrequent smokers are nondaily smokers who smoked on ≤8 days in the past 30 days.

^g^Lifetime abstainers had fewer than 12 drinks in lifetime; Former drinkers had at least 12 drinks in lifetime, but none in past year; Current light drinkers drank 1–3 drinks per week in past year; Current moderate drinkers drank 4–14 drinks per week for male and 4–7 drinks per week for female; Current heavy drinkers drank >14 drinks per week for male and >7 drinks per week for female.

^h^Poverty status is a ratio of family income to the appropriate poverty threshold (given family size and number of children) defined by the US Census Bureau. “Poor” adults reported a family income below the poverty threshold. “Near poor” adults had a family income of 100–199% of the poverty threshold. “Not poor” adults reported a family income of 200% of the poverty threshold or greater.

*P < .05;

**P < .01;

***P < .001

Smokefree bar law coverage yielded similar results (S2 Table): Stronger smokefree bar law coverage was associated with less current smoking (OR = 0.84, *p* = .015), but was not associated with any measures of LITS among smokers. Increased drinking frequency was related to increased odds of current smoking, but was not related to LITS patterns. Though the interaction of smokefree bar law coverage and drinking frequency was significant (*p* = .013), this isolated result could be a false positive due to the large number of comparisons.

Among alcohol drinkers, higher smokefree law coverage (OR = 0.79, *p* = .023) and bar law coverage (OR = 0.84, *p* = .027) were associated with lower odds of current smoking, but not with any LITS patterns (S3 and S4 Tables). Binge drinking increased the odds of current smoking, but was unrelated to LITS.

### Smokefree law coverage, alcohol use, and smoking quit attempts

Among current smokers, smokefree law coverage and bar law coverage were not associated with quit attempts among any of the 6 smoking subgroups (Table 3 and S5 Table). Alcohol use was not related to smoking quit attempts generally. None of the interactions were significant. Among drinkers who reported each of the 6 smoking patterns, smokefree law (and bar law) coverage and binge drinking were not associated with quit attempts (S6 and S7 Tables).

**Table 3:** Relationship of smokefree law coverage and alcohol use with smoking quit attempts. *Note*. AOR = adjusted odds ratio; CI = confidence interval.

Subpopulation	Current smoker^a^	Daily smoker^b^	Nondaily smoker^c^	Very light daily smoker^d^	Very light nondaily smoker^e^	Infrequent smoker^f^
	AOR (95% CI)	AOR (95% CI)	AOR (95% CI)	AOR (95% CI)	AOR (95% CI)	AOR (95% CI)
N	5431	4306	1099	705	649	408
**Smokefree law coverage score**	0.94 (0.69, 1.29)	0.99 (0.70, 1.40)	0.85 (0.46, 1.60)	0.83 (0.34, 2.01)	1.03 (0.48, 2.20)	0.76 (0.27, 2.16)
**Drinking status** ^g^						
Lifetime abstainer	1.01 (0.67, 1.53)	1.01 (0.65, 1.58)	1.20 (0.43, 3.31)	0.89 (0.26, 3.00)	1.25 (0.29, 5.37)	0.44 (0.09, 2.13)
Former drinker	0.88 (0.64, 1.20)	0.92 (0.65, 1.30)	0.99 (0.35, 2.75)	0.57 (0.17, 1.86)	0.96 (0.26, 3.60)	9.22 (0.78, 108.54)
Current light drinker	1.00	1.00	1.00	1.00	1.00	1.00
Current moderate drinker	0.80 (0.59, 1.10)	0.81 (0.55, 1.19)	0.75 (0.37, 1.52)	0.39 (0.13, 1.15)	1.48 (0.59, 3.75)	1.05 (0.33, 3.30)
Current heavy drinker	0.72 (0.47, 1.08)	0.75 (0.48, 1.17)	0.86 (0.29, 2.51)	0.58 (0.14, 2.38)	1.33 (0.37, 4.79)	0.25 (0.05, 1.34)
**Age group (years)**						
18–20	1.41 (0.95, 2.08)	1.52 (0.93, 2.49)	0.78 (0.39, 1.58)	1.39 (0.51, 3.78)	0.72 (0.28, 1.81)	0.56 (0.16, 1.97)
21–24	1.73 (1.26, 2.36)**	1.88 (1.29, 2.73)**	1.08 (0.62, 1.87)	1.57 (0.79, 3.12)	1.08 (0.54, 2.15)	1.29 (0.46, 3.61)
25–44	1.17 (1.00, 1.37)*	1.12 (0.93, 1.34)	1.30 (0.90, 1.88)	1.25 (0.78, 2.01)	1.29 (0.79, 2.10)	0.91 (0.47, 1.77)
45–64	1.00	1.00	1.00	1.00	1.00	1.00
65 and above	0.88 (0.68, 1.13)	0.80 (0.60, 1.06)	1.11 (0.60, 2.08)	0.72 (0.34, 1.50)	1.00 (0.41, 2.44)	1.23 (0.45, 3.33)
**Female**	1.06 (0.91, 1.23)	1.01 (0.85, 1.19)	1.29 (0.96, 1.73)	1.15 (0.76, 1.74)	1.90 (1.24, 2.91)**	1.66 (0.97, 2.84)
**Race/ethnicity**						
White, non-Hispanic	1.00	1.00	1.00	1.00	1.00	1.00
Black, non-Hispanic	1.38 (1.14, 1.68)**	1.39 (1.13, 1.71)**	1.05 (0.61, 1.81)	0.84 (0.51, 1.38)	0.76 (0.39, 1.48)	2.34 (0.93, 5.85)
API and others, non-Hispanic	1.25 (0.89, 1.75)	1.11 (0.76, 1.61)	1.71 (0.82, 3.57)	0.55 (0.27, 1.11)	1.26 (0.47, 3.34)	2.84 (0.88, 9.18)
Hispanic	1.39 (1.07, 1.81)*	1.51 (1.08, 2.10)*	0.69 (0.45, 1.06)	0.70 (0.41, 1.21)	0.68 (0.40, 1.18)	1.44 (0.76, 2.73)
**Education**						
0–12 years (no diploma)	0.80 (0.62, 1.01)	0.78 (0.57, 1.05)	1.89 (1.09, 3.30)*	1.10 (0.54, 2.24)	3.01 (1.44, 6.30)**	2.35 (0.97, 5.71)
High school graduate/GED	0.94 (0.76, 1.16)	0.97 (0.74, 1.26)	1.47 (0.90, 2.42)	1.20 (0.64, 2.25)	2.13 (1.05, 4.31)*	0.96 (0.39, 2.38)
Some college (no diploma)/associate degree	1.15 (0.93, 1.43)	1.19 (0.90, 1.56)	1.44 (0.96, 2.15)	1.59 (0.84, 3.01)	1.42 (0.85, 2.37)	1.19 (0.62, 2.29)
Undergraduate/graduate degree	1.00	1.00	1.00	1.00	1.00	1.00
**Poverty status** ^h^						
<100% (Poor)	0.93 (0.77, 1.13)	0.96 (0.77, 1.20)	0.95 (0.59, 1.53)	1.07 (0.64, 1.76)	0.70 (0.40, 1.21)	0.59 (0.28, 1.26)
100–199% (Near poor)	0.97 (0.82, 1.15)	1.01 (0.83, 1.22)	0.95 (0.61, 1.48)	1.24 (0.76, 2.03)	0.96 (0.53, 1.74)	0.97 (0.46, 2.04)
≥200% (Not poor)	1.00	1.00	1.00	1.00	1.00	1.00
Unspecified	0.93 (0.71, 1.23)	0.98 (0.71, 1.34)	0.85 (0.49, 1.49)	1.09 (0.58, 2.04)	1.36 (0.56, 3.32)	0.92 (0.41, 2.07)
**Cigarette pack price (US dollar)**	1.12 (1.01, 1.24)*	1.09 (0.97, 1.23)	1.22 (0.98, 1.52)	1.19 (0.95, 1.51)	1.40 (1.07, 1.84)*	1.23 (0.86, 1.74)
**Smokefree law coverage × drinking status**	F_(4, 297)_ = 0.14; *p* = .969	F_(4, 297)_ = 0.20; *p* = .939	F_(4, 282)_ = 0.37; *p* = .831	F_(4, 252)_ = 0.97; *p* = .426	F_(4, 256)_ = 0.46; *p* = .762	F_(4, 206)_ = 1.28; *p* = .278

^a^Current smokers smoked at least 100 cigarettes in their lifetime and smoked “every day” or “some days” now.

^b^Daily smokers smoked “every day” now, or if they smoked “some days,” they smoked on >25 days in the past 30 days.

^c^Nondaily smokers smoked “some days” now and smoked on ≤25 days in the past 30 days.

^d^Very light daily smokers are daily smokers who smoked ≤5 cigarettes per day.

^e^Very light nondaily smokers are nondaily smokers who smoked ≤3 cigarettes per day.

^f^Infrequent smokers are nondaily smokers who smoked on ≤8 days in the past 30 days.

^g^Lifetime abstainers had fewer than 12 drinks in lifetime; Former drinkers had at least 12 drinks in lifetime, but none in past year; Current light drinkers drank 1–3 drinks per week in past year; Current moderate drinkers drank 4–14 drinks per week for male and 4–7 drinks per week for female; Current heavy drinkers drank >14 drinks per week for male and >7 drinks per week for female.

^h^Poverty status is a ratio of family income to the appropriate poverty threshold (given family size and number of children) defined by the US Census Bureau. “Poor” adults reported a family income below the poverty threshold. “Near poor” adults had a family income of 100–199% of the poverty threshold. “Not poor” adults reported a family income of 200% of the poverty threshold or greater.

*P < .05;

**P < .01.

## Discussion

This is the first study to examine the relationship of smokefree law (and bar law) coverage and alcohol use with current smoking, LITS, and smoking quit attempts in a large population-based sample of US adults and alcohol drinkers. Stronger smokefree law and bar law coverage was associated with reduced odds of current smoking, but was not related to any measure of LITS or smoking quit attempts. The association between smokefree law coverage and current smoking did not vary by drinking or binge drinking status.

Consistent with earlier reports of a negative relationship between smokefree law coverage and current smoking among adults [4,5], we found that the relationship was also observed among alcohol drinkers. Stronger smokefree law (and bar law) coverage not only benefited the general adult population in reducing the odds of current smoking, but also benefited the alcohol drinkers who were at high risk for tobacco use. The relationship between smokefree law (and bar law) coverage and current smoking was similar across all drinking and binge drinking subgroups, indicating that the laws benefited all drinking subgroups equally without creating smoking disparities. Longitudinal study is needed to investigate how drinkers at different drinking and binge drinking levels respond to smokefree laws in changing their smoking behaviors.

Though published literature concluded that smokefree law coverage was associated with lower cigarette consumption [4], we found smokefree law coverage was not associated with any LITS patterns among smokers and among current drinking smokers. Our finding might be explained by the fact that smokefree laws have more effect on heavy smokers than light and intermittent smokers, and the reduction in cigarette consumption among heavier smokers were not large enough to switch them to light and intermittent smokers by our definitions. This explanation is consistent with Borland et al.’s [40] finding that smokefree workplaces reduced cigarette consumption by 7.9 cig/day for heavy smokers, 5.8 cig/day for moderate smokers, but had no effect on light smokers (<15 cig/day). Woodruff et al. [41] found that smokefree workplaces reduced cigarette consumption by 45 packs per year (≈2.5 cig/day) among regular smokers; Dinno and Glantz [4] reported that strong smokefree laws were associated with a reduction of 2.36 cig/day among continuing smokers. Future studies need to take into account the different types of smokers while investigating the effect of smokefree laws on cigarette consumptions as these smokers may respond differently to the laws.

Smokefree law and bar law coverage was not associated with smoking quit attempts among any smoking subgroups or drinkers who reported different smoking patterns. One prior study found that the association between smokefree restaurant and bar laws and smoking quit attempts varied depending on the smoker’s gender and diagnosis of mental illness [22], specifically the laws were associated with more quit attempts only among males without mental illness, men with alcohol use disorders, and females with anxiety disorders [22]. Other studies found smokers who worked in a smokefree worksite [6,42] or live in towns with 100% smokefree restaurant laws were more likely to report quit attempts [8]. While these studies used different methods with the present study to calculate the law coverage, their findings indicated that some factors such as gender and mental illness could moderate the relationship between smokefree laws and smoking quit attempts.

Increased alcohol use and binge drinking were associated with current smoking, but were not associated with any LITS patterns or smoking quit attempts for any smoking subgroups. Our findings were partly consistent with prior study [9] which found that drinking frequency was not associated with smoking quit attempts among current smokers and smokers such as occasional, regular, very light, heavier smokers. But binge drinking was negatively associated with quit attempts among very light smokers [9]. The different findings on the relationship between alcohol use and quit attempts might be related to the study population (general adults and drinkers vs. young adult bar patrons). Future research needs to consider the different types of smokers (especially light and intermittent smokers as LITS has become increasingly common) while investigating the effect of alcohol consumption on smoking quit attempts.

This is cross-sectional study so we cannot make conclusions about the causality between smokefree law coverage and smoking status or quit attempts. Smoking and drinking data were based on self-report, without biochemical validation, and were subject to recall bias. The response rate was 65.4%, which, while not ideal, is acceptable by current standards. We classified cigarette consumption into different levels and treated it as categorical variable. Therefore we did not observe a dose-related association between LITS and smokefree law coverage or alcohol consumption. Despite the limitations, we found that stronger smokefree law (and bar law) coverage was associated with decreased odds of current smoking among the general US adult population and among alcohol drinkers. The effect of the laws was same across all adults without creating smoking disparities based on the respondents’ drinking and binge drinking status. This finding provides further support for these public health policies. However, among remaining smokers, the smokefree law coverage was not associated with any LITS patterns or smoking quit attempts. To encourage more smokers to try quitting, or help heavy daily smokers further reduce cigarette consumption and switch to light and intermittent smokers, strict smokefree laws (e.g., extension of smokefree laws to outdoor areas) combined with more other tobacco control measures may be considered.

## Supporting Information

S1 Table Sample characteristics. (DOCX)

S2 Table Relationship of smokefree bar law coverage and alcohol use with smoking. (DOCX)

S3 Table Relationship of smokefree law coverage and alcohol use with smoking among current drinkers. (DOCX)

S4 Table Relationship of smokefree bar law coverage and alcohol use with smoking among current drinkers. (DOCX)

S5 Table Relationship of smokefree bar law coverage and alcohol use with smoking quit attempts. (DOCX)

S6 Table Relationship of smokefree law coverage and drinking with smoking quit attempts among current drinking smokers. (DOCX)

S7 Table Relationship of smokefree bar law coverage and drinking with smoking quit attempts among drinking smokers. (DOCX)
