# Supplementary material for: Relationship of Smokefree Laws and Alcohol Use with Light and Intermittent Smoking and Quit Attempts among US Adults and Alcohol Users
Source: PLoS One. 2015 Oct 7;10(10):e0137023. doi: 10.1371/journal.pone.0137023 (PMC4596828; doi:10.1371/journal.pone.0137023)
Supplement: S1 Table — (DOCX) [file pone.0137023.s001.docx]

**Supporting information**

**Relationship of Smokefree Laws and Alcohol Use with Light and Intermittent Smoking and Quit Attempts among US Adults and Alcohol Users**

Nan Jiang, MariaElena Gonzalez, Pamela M. Ling, Stanton A. Glantz

**S1 Table. Sample characteristics**

|  | | n | % [95% CI] |
| --- | --- | --- | --- |
| **Total** | | **27731** | **100%** |
| **Age group (years)** | |  |  |
| 18-20 | | 1059 | 5.1 [4.6, 5.6] |
| 21-24 | | 1763 | 7.7 [7.1, 8.2] |
| 25-44 | | 9953 | 35.8 [34.9, 36.7] |
| 45-64 | | 9463 | 34.8 [34.0, 35.7] |
| 65 and above | | 5493 | 16.6 [16.0, 17.3] |
| **Gender** | |  |  |
| Male | | 12261 | 48.3 [47.6, 49.0] |
| Female | | 15470 | 51.7 [51.0, 52.4] |
| **Race/ethnicity** | |  |  |
| White, non-Hispanic | | 16268 | 68.8 [67.8, 69.8] |
| Black, non-Hispanic | | 4464 | 11.9 [11.2, 12.5] |
| API and others, non-Hispanic | | 1834 | 5.6 [5.1, 6.0] |
| Hispanic | | 5165 | 13.8 [13.1, 14.4] |
| **Education** | |  |  |
| 0-12 years (no diploma) | | 4759 | 14.8 [14.2, 15.5] |
| High school graduate/GED | | 7463 | 27.8 [27.0, 28.6] |
| Some college (no degree) or associate degree | | 8217 | 30.5 [29.7, 31.2] |
| Undergraduate/graduate degree | | 7147 | 26.9 [26.0, 27.8] |
| **Poverty status^a^** | |  |  |
| <100% (Poor) | | 4341 | 11.7 [11.1, 12.2] |
| 100-199% (Near poor) | | 4718 | 15.3 [14.8, 15.9] |
| ≥200% (Not poor) | | 15407 | 60.9 [60.0, 61.7] |
| Unspecified | | 3265 | 12.2 [11.5, 12.8] |
| **Drinking status^b^** | |  |  |
| Lifetime abstainer | | 5910 | 20.0 [19.2, 20.8] |
| Former drinker | | 4336 | 15.0 [14.4, 15.7] |
| Current light drinker | | 11554 | 43.9 [43.1, 44.7] |
| Current moderate drinker | | 4005 | 15.7 [15.1, 16.4] |
| Current heavy drinker | | 1402 | 5.3 [4.9, 5.7] |
| **Binge drinking^c^ (among current drinkers)** | |  |  |
| No | | 11055 | 41.7 [40.8, 42.6] |
| Yes | | 5746 | 23.0 [22.2, 23.9] |
| **Smokefree law coverage score** | 0.63 | | |
| **Smokefree bar law coverage score** | 0.52 | | |
| **Cigarette pack price (US dollar)** | 4.76 | | |

*Note.*

^a^Poverty status is a ratio of family income to the appropriate poverty threshold (given family size and number of children) defined by the US Census Bureau. “Poor” adults reported a family income below the poverty threshold. “Near poor” adults had a family income of 100-199% of the poverty threshold. “Not poor” adults reported a family income of 200% of the poverty threshold or greater.

^b^Lifetime abstainers had fewer than 12 drinks in lifetime; Former drinkers had at least 12 drinks in lifetime, but none in past year; Current light drinkers drank 1-3 drinks per week in past year; Current moderate drinkers drank 4-14 drinks per week for male and 4-7 drinks per week for female; Current heavy drinkers drank >14 drinks per week for male and >7 drinks per week for female.

^c^Binge drinkers drank ≥5 drinks on at least one day in the past 12 months.
