# Supplementary material for: Relationship of Smokefree Laws and Alcohol Use with Light and Intermittent Smoking and Quit Attempts among US Adults and Alcohol Users
Source: PLoS One. 2015 Oct 7;10(10):e0137023. doi: 10.1371/journal.pone.0137023 (PMC4596828; doi:10.1371/journal.pone.0137023)
Supplement: S2 Table — (DOCX) [file pone.0137023.s002.docx]

**Supporting information**

**Relationship of Smokefree Laws and Alcohol Use with Light and Intermittent Smoking and Quit Attempts among US Adults and Alcohol Users**

Nan Jiang, MariaElena Gonzalez, Pamela M. Ling, Stanton A. Glantz

**S2 Table. Relationship of smokefree bar law coverage and alcohol use with smoking**

| Subpopulation | Adult | Current smoker^a^ | Daily smoker^b^ | Nondaily smoker^c^ | |
| --- | --- | --- | --- | --- | --- |
| Outcome | Current smoker | Nondaily smoker | Very light daily smoker^d^ | Very light nondaily smoker^e^ | Infrequent smoker^f^ |
|  | AOR (95% CI) | AOR (95% CI) | AOR (95% CI) | AOR (95% CI) | AOR (95% CI) |
| N | 27055 | 5411 | 4289 | 1047 | 1101 |
| **Smokefree bar law coverage score** | 0.84 (0.73, 0.97)* | 1.01 (0.76, 1.33) | 1.13 (0.79, 1.62) | 1.02 (0.60, 1.72) | 0.87 (0.51, 1.47) |
| **Drinking status^g^** |  |  |  |  |  |
| Lifetime abstainer | 0.36 (0.30, 0.43)*** | 0.77 (0.48, 1.23) | 1.50 (0.94, 2.39) | 0.98 (0.37, 2.59) | 1.28 (0.60, 2.72) |
| Former drinker | 1.01 (0.84, 1.21) | 0.56 (0.36, 0.86)** | 0.97 (0.65, 1.47) | 0.45 (0.21, 0.97)* | 0.97 (0.40, 2.39) |
| Current light drinker | 1.00 | 1.00 | 1.00 | 1.00 | 1.00 |
| Current moderate drinker | 1.60 (1.35, 1.90)*** | 1.20 (0.88, 1.65) | 1.03 (0.66, 1.58) | 0.89 (0.48, 1.68) | 0.83 (0.43, 1.60) |
| Current heavy drinker | 3.77 (3.01, 4.73)*** | 0.84 (0.53, 1.35) | 1.51 (0.83, 2.73) | 0.27 (0.12, 0.60)** | 0.91 (0.35, 2.37) |
| **Age group (years)** |  |  |  |  |  |
| 18-20 | 0.82 (0.64, 1.06) | 3.10 (1.95, 4.94)*** | 3.37 (1.92, 5.92)*** | 2.84 (1.31, 6.18)** | 0.43 (0.18, 1.01) |
| 21-24 | 0.99 (0.82, 1.19) | 1.99 (1.41, 2.81)*** | 2.09 (1.37, 3.19)** | 2.45 (1.31, 4.57)** | 1.08 (0.62, 1.90) |
| 25-44 | 1.29 (1.17, 1.42)*** | 1.51 (1.23, 1.86)*** | 1.01 (0.79, 1.29) | 1.27 (0.86, 1.87) | 0.96 (0.66, 1.40) |
| 45-64 | 1.00 | 1.00 | 1.00 | 1.00 | 1.00 |
| 65 and above | 0.32 (0.28, 0.38)*** | 1.38 (0.97, 1.96) | 1.28 (0.89, 1.84) | 0.72 (0.35, 1.49) | 1.30 (0.67, 2.50) |
| **Female** | 0.86 (0.79, 0.93)*** | 0.95 (0.79, 1.14) | 1.45 (1.13, 1.86)** | 1.27 (0.92, 1.77) | 0.97 (0.70, 1.35) |
| **Race/ethnicity** |  |  |  |  |  |
| White, non-Hispanic | 1.00 | 1.00 | 1.00 | 1.00 | 1.00 |
| Black, non-Hispanic | 0.76 (0.68, 0.86)*** | 1.64 (1.27, 2.13)*** | 3.54 (2.70, 4.64)*** | 1.36 (0.85, 2.18) | 0.77 (0.47, 1.25) |
| API and others, non-Hispanic | 0.89 (0.73, 1.09) | 1.34 (0.87, 2.07) | 3.38 (2.13, 5.38)*** | 0.95 (0.43, 2.09) | 0.84 (0.42, 1.67) |
| Hispanic | 0.36 (0.31, 0.41)*** | 2.77 (2.10, 3.64)*** | 6.98 (5.23, 9.30)*** | 2.94 (1.82, 4.76)*** | 1.72 (1.12, 2.65)* |
| **Education** |  |  |  |  |  |
| 0-12 years (no diploma) | 6.16 (5.20, 7.31)*** | 0.26 (0.19, 0.37)*** | 0.47 (0.31, 0.70)*** | 0.72 (0.43, 1.20) | 0.76 (0.42, 1.39) |
| High school graduate/GED | 4.67 (4.08, 5.34)*** | 0.32 (0.25, 0.43)*** | 0.52 (0.36, 0.76)** | 1.11 (0.70, 1.76) | 0.49 (0.29, 0.82)** |
| Some college (no diploma)/associate degree | 2.75 (2.43, 3.11)*** | 0.47 (0.36, 0.61)*** | 0.58 (0.40, 0.83)** | 1.36 (0.85, 2.18) | 0.65 (0.42, 0.99)* |
| Undergraduate/graduate degree | 1.00 | 1.00 | 1.00 | 1.00 | 1.00 |
| **Poverty status^h^** |  |  |  |  |  |
| <100% (Poor) | 1.95 (1.73, 2.21)*** | 0.82 (0.66, 1.04) | 1.11 (0.82, 1.49) | 0.55 (0.35, 0.85)** | 0.89 (0.54, 1.47) |
| 100-199% (Near poor) | 1.48 (1.31, 1.67)*** | 0.79 (0.63, 0.99)* | 0.99 (0.74, 1.33) | 1.21 (0.79, 1.87) | 0.88 (0.58, 1.32) |
| ≥200% (Not poor) | 1.00 | 1.00 | 1.00 | 1.00 | 1.00 |
| Unspecified | 1.01 (0.88, 1.17) | 0.90 (0.64, 1.28) | 1.26 (0.88, 1.79) | 0.76 (0.40, 1.44) | 1.47 (0.83, 2.62) |
| **Cigarette pack price (US dollar)** | 1.04 (0.97, 1.10) | 0.97 (0.87, 1.08) | 1.12 (0.96, 1.30) | 1.15 (0.93, 1.43) | 1.08 (0.87, 1.33) |
| **Smokefree bar law coverage × drinking status** | F_(4, 297)_=3.25; *p*=.013 | F_(4, 297)_=0.85; *p*=.497 | F_(4, 297)_=1.79; *p*=.130 | F_(4, 281)_=1.06; *p*=.375 | F_(4, 282)_=0.34; *p*=.851 |

*Note.* AOR=adjusted odds ratio; CI=confidence interval.

^a^Current smokers smoked at least 100 cigarettes in their lifetime and smoked “every day” or “some days” now.

^b^Daily smokers smoked “every day” now, or if they smoked “some days”, they smoked on >25 days in the past 30 days.

^c^Nondaily smokers smoked “some days” now and smoked on ≤25 days in the past 30 days.

^d^Very light daily smokers are daily smokers who smoked ≤5 cigarettes per day.

^e^Very light nondaily smokers are nondaily smokers who smoked ≤3 cigarettes per day.

^f^Infrequent smokers are nondaily smokers who smoked on ≤8 days in the past 30 days.

^g^Lifetime abstainers had fewer than 12 drinks in lifetime; Former drinkers had at least 12 drinks in lifetime, but none in past year; Current light drinkers drank 1-3 drinks per week in past year; Current moderate drinkers drank 4-14 drinks per week for male and 4-7 drinks per week for female; Current heavy drinkers drank >14 drinks per week for male and >7 drinks per week for female.

^h^Poverty status is a ratio of family income to the appropriate poverty threshold (given family size and number of children) defined by the US Census Bureau. “Poor” adults reported a family income below the poverty threshold. “Near poor” adults had a family income of 100-199% of the poverty threshold. “Not poor” adults reported a family income of 200% of the poverty threshold or greater.

^*^*P*<.05; ^**^*P*<.01; ^***^*P*<.001.
