# Supplementary material for: Relationship of Smokefree Laws and Alcohol Use with Light and Intermittent Smoking and Quit Attempts among US Adults and Alcohol Users
Source: PLoS One. 2015 Oct 7;10(10):e0137023. doi: 10.1371/journal.pone.0137023 (PMC4596828; doi:10.1371/journal.pone.0137023)
Supplement: S4 Table — (DOCX) [file pone.0137023.s004.docx]

**Supporting information**

**Relationship of Smokefree Laws and Alcohol Use with Light and Intermittent Smoking and Quit Attempts among US Adults and Alcohol Users**

Nan Jiang, MariaElena Gonzalez, Pamela M. Ling, Stanton A. Glantz

**S4 Table. Relationship of smokefree bar law coverage and alcohol use with smoking among current drinkers**

| Subpopulation | Adult | Current smoker^a^ | Daily smoker^b^ | Nondaily smoker^c^ | |
| --- | --- | --- | --- | --- | --- |
| Outcome | Current smoker | Nondaily smoker | Very light daily smoker^d^ | Very light nondaily smoker^e^ | Infrequent smoker^f^ |
|  | AOR (95% CI) | AOR (95% CI) | AOR (95% CI) | AOR (95% CI) | AOR (95% CI) |
| N | 16734 | 3929 | 3023 | 856 | 899 |
| **Smokefree bar law coverage score** | 0.84 (0.72, 0.98)* | 0.94 (0.69, 1.28) | 0.99 (0.67, 1.45) | 1.02 (0.55, 1.90) | 0.89 (0.50, 1.57) |
| **Binge drinking^g^** |  |  |  |  |  |
| No | 1.00 | 1.00 | 1.00 | 1.00 | 1.00 |
| Yes | 2.27 (1.94, 2.67)*** | 0.93 (0.68, 1.27) | 1.00 (0.67, 1.49) | 0.76 (0.45, 1.30) | 1.00 (0.56, 1.80) |
| **Age group (years)** |  |  |  |  |  |
| 18-20 | 0.67 (0.48, 0.94)* | 2.04 (1.21, 3.45)** | 4.08 (1.94, 8.58)*** | 2.49 (0.95, 6.51) | 0.75 (0.28, 2.02) |
| 21-24 | 0.86 (0.68, 1.08) | 2.13 (1.44, 3.17)*** | 2.53 (1.61, 3.97)*** | 2.47 (1.26, 4.84)** | 1.11 (0.59, 2.09) |
| 25-44 | 1.19 (1.05, 1.33)** | 1.57 (1.24, 1.97)*** | 1.31 (0.99, 1.72) | 1.25 (0.82, 1.92) | 1.09 (0.71, 1.67) |
| 45-64 | 1.00 | 1.00 | 1.00 | 1.00 | 1.00 |
| 65 and above | 0.40 (0.32, 0.49)*** | 1.52 (0.94, 2.46) | 1.89 (1.14, 3.13)* | 0.51 (0.23, 1.13) | 1.38 (0.52, 3.71) |
| **Female** | 0.93 (0.84, 1.03) | 0.89 (0.72, 1.10) | 1.60 (1.20, 2.12)** | 1.24 (0.86, 1.80) | 0.98 (0.68, 1.42) |
| **Race/ethnicity** |  |  |  |  |  |
| White, non-Hispanic | 1.00 | 1.00 | 1.00 | 1.00 | 1.00 |
| Black, non-Hispanic | 0.86 (0.74, 1.01) | 1.65 (1.23, 2.21)** | 3.74 (2.68, 5.23)*** | 1.54 (0.90, 2.64) | 0.76 (0.44, 1.33) |
| API and others, non-Hispanic | 1.01 (0.80, 1.28) | 1.26 (0.78, 2.03) | 3.70 (2.24, 6.10)*** | 1.26 (0.49, 3.25) | 0.87 (0.39, 1.91) |
| Hispanic | 0.37 (0.32, 0.44)*** | 3.13 (2.26, 4.33)*** | 8.06 (5.55, 11.70)*** | 2.78 (1.62, 4.75)*** | 1.95 (1.19, 3.18)** |
| **Education** |  |  |  |  |  |
| 0-12 years (no diploma) | 6.80 (5.55, 8.32)*** | 0.23 (0.16, 0.32)*** | 0.44 (0.27, 0.70)** | 1.17 (0.61, 2.23) | 0.45 (0.25, 0.80)** |
| High school graduate/GED | 4.59 (3.97, 5.31)*** | 0.30 (0.22, 0.41)*** | 0.55 (0.37, 0.83)** | 0.74 (0.42, 1.29) | 0.65 (0.41, 1.03) |
| Some college (no diploma)/associate degree | 2.67 (2.35, 3.04)*** | 0.47 (0.35, 0.62)*** | 0.63 (0.42, 0.95)* | 1.20 (0.74, 1.96) | 0.76 (0.44, 1.33) |
| Undergraduate/graduate degree | 1.00 | 1.00 | 1.00 | 1.00 | 1.00 |
| **Poverty status^h^** |  |  |  |  |  |
| <100% (Poor) | 1.91 (1.61, 2.25)*** | 0.89 (0.68, 1.15) | 0.96 (0.67, 1.40) | 0.51 (0.32, 0.83)** | 0.76 (0.43, 1.33) |
| 100-199% (Near poor) | 1.57 (1.34, 1.83)*** | 0.70 (0.53, 0.91)** | 0.89 (0.63, 1.27) | 1.31 (0.79, 2.18) | 0.82 (0.51, 1.32) |
| ≥200% (Not poor) | 1.00 | 1.00 | 1.00 | 1.00 | 1.00 |
| Unspecified | 1.06 (0.88, 1.27) | 0.93 (0.62, 1.38) | 1.40 (0.89, 2.20) | 0.60 (0.30, 1.20) | 1.47 (0.74, 2.91) |
| **Cigarette pack price (US dollar)** | 0.98 (0.91, 1.05) | 0.96 (0.85, 1.09) | 1.10 (0.94, 1.28) | 1.09 (0.86, 1.39) | 1.01 (0.80, 1.28) |
| **Smokefree bar law coverage × binge drinking** | F_(1, 300)_=0.01; *p*=.941 | F_(1, 300)_=3.95; *p*=.048 | F_(1, 299)_=0.45; *p*=.504 | F_(1, 271)_=0.10; *p*=.752 | F_(1, 273)_=0.47; *p*=.492 |

*Note.* AOR=adjusted odds ratio; CI=confidence interval.

^a^Current smokers smoked at least 100 cigarettes in their lifetime and smoked “every day” or “some days” now.

^b^Daily smokers smoked “every day” now, or if they smoked “some days”, they smoked on >25 days in the past 30 days.

^c^Nondaily smokers smoked “some days” now and smoked on ≤25 days in the past 30 days.

^d^Very light daily smokers are daily smokers who smoked ≤5 cigarettes per day.

^e^Very light nondaily smokers are nondaily smokers who smoked ≤3 cigarettes per day.

^f^Infrequent smokers are nondaily smokers who smoked on ≤8 days in the past 30 days.

^g^Binge drinkers drank ≥5 drinks on at least one day in the past 12 months.

^h^Poverty status is a ratio of family income to the appropriate poverty threshold (given family size and number of children) defined by the US Census Bureau. “Poor” adults reported a family income below the poverty threshold. “Near poor” adults had a family income of 100-199% of the poverty threshold. “Not poor” adults reported a family income of 200% of the poverty threshold or greater.

^*^*P*<.05; ^**^*P*<.01; ^***^*P*<.001.
