# Supplementary material for: Relationship of Smokefree Laws and Alcohol Use with Light and Intermittent Smoking and Quit Attempts among US Adults and Alcohol Users
Source: PLoS One. 2015 Oct 7;10(10):e0137023. doi: 10.1371/journal.pone.0137023 (PMC4596828; doi:10.1371/journal.pone.0137023)
Supplement: S5 Table — (DOCX) [file pone.0137023.s005.docx]

**Supporting information**

**Relationship of Smokefree Laws and Alcohol Use with Light and Intermittent Smoking and Quit Attempts among US Adults and Alcohol Users**

Nan Jiang, MariaElena Gonzalez, Pamela M. Ling, Stanton A. Glantz

**S5 Table. Relationship of smokefree bar law coverage and alcohol use with smoking quit attempts**

| Subpopulation | Current smoker^a^ | Daily smoker^b^ | Nondaily smoker^c^ | Very light daily smoker^d^ | Very light nondaily smoker^e^ | Infrequent smoker^f^ |
| --- | --- | --- | --- | --- | --- | --- |
|  | AOR (95% CI) | AOR (95% CI) | AOR (95% CI) | AOR (95% CI) | AOR (95% CI) | AOR (95% CI) |
| N | 5431 | 4306 | 1099 | 705 | 649 | 408 |
| **Smokefree bar law coverage score** | 1.07 (0.84, 1.37) | 1.11 (0.84, 1.46) | 1.04 (0.64, 1.69) | 0.89 (0.46, 1.71) | 1.48 (0.80, 2.76) | 0.95 (0.43, 2.10) |
| **Drinking status^g^** |  |  |  |  |  |  |
| Lifetime abstainer | 1.07 (0.81, 1.44) | 1.14 (0.82, 1.57) | 0.98 (0.44, 2.20) | 1.02 (0.46, 2.28) | 1.02 (0.35, 3.03) | 0.44 (0.15, 1.28) |
| Former drinker | 0.95 (0.75, 1.21) | 1.01 (0.77, 1.31) | 0.98 (0.45, 2.11) | 0.70 (0.30, 1.67) | 0.98 (0.33, 2.86) | 6.08 (1.23, 30.14)* |
| Current light drinker | 1.00 | 1.00 | 1.00 | 1.00 | 1.00 | 1.00 |
| Current moderate drinker | 0.80 (0.62, 1.02) | 0.78 (0.59, 1.04) | 0.81 (0.46, 1.43) | 0.44 (0.21, 0.95)* | 1.70 (0.83, 3.50) | 1.16 (0.47, 2.88) |
| Current heavy drinker | 0.78 (0.57, 1.07) | 0.81 (0.58, 1.12) | 0.81 (0.37, 1.79) | 0.58 (0.21, 1.58) | 1.03 (0.37, 2.87) | 0.33 (0.08, 1.27) |
| **Age group (years)** |  |  |  |  |  |  |
| 18-20 | 1.39 (0.94, 2.06) | 1.50 (0.92, 2.45) | 0.77 (0.38, 1.57) | 1.36 (0.50, 3.69) | 0.76 (0.30, 1.93) | 0.49 (0.15, 1.67) |
| 21-24 | 1.73 (1.27, 2.36)** | 1.88 (1.30, 2.73)** | 1.10 (0.64, 1.90) | 1.54 (0.77, 3.06) | 1.11 (0.57, 2.17) | 1.34 (0.49, 3.62) |
| 25-44 | 1.17 (1.00, 1.37)* | 1.12 (0.93, 1.34) | 1.32 (0.91, 1.92) | 1.24 (0.77, 2.00) | 1.34 (0.82, 2.19) | 0.91 (0.47, 1.75) |
| 45-64 | 1.00 | 1.00 | 1.00 | 1.00 | 1.00 | 1.00 |
| 65 and above | 0.88 (0.68, 1.13) | 0.80 (0.60, 1.06) | 1.12 (0.60, 2.09) | 0.72 (0.35, 1.50) | 1.01 (0.42, 2.45) | 1.22 (0.46, 3.22) |
| **Female** | 1.05 (0.91, 1.23) | 1.00 (0.85, 1.19) | 1.28 (0.95, 1.72) | 1.13 (0.75, 1.72) | 1.93 (1.26, 2.94)** | 1.67 (0.98, 2.84) |
| **Race/ethnicity** |  |  |  |  |  |  |
| White, non-Hispanic | 1.00 | 1.00 | 1.00 | 1.00 | 1.00 | 1.00 |
| Black, non-Hispanic | 1.38 (1.14, 1.68)** | 1.40 (1.14, 1.72)** | 1.02 (0.60, 1.74) | 0.87 (0.53, 1.44) | 0.76 (0.39, 1.47) | 2.11 (0.84, 5.34) |
| API and others, non-Hispanic | 1.23 (0.87, 1.73) | 1.10 (0.75, 1.61) | 1.67 (0.79, 3.56) | 0.55 (0.27, 1.11) | 1.20 (0.45, 3.16) | 2.79 (0.84, 9.28) |
| Hispanic | 1.38 (1.06, 1.79)* | 1.50 (1.08, 2.08)* | 0.69 (0.45, 1.07) | 0.70 (0.41, 1.20) | 0.69 (0.39, 1.20) | 1.44 (0.77, 2.70) |
| **Education** |  |  |  |  |  |  |
| 0-12 years (no diploma) | 0.80 (0.63, 1.02) | 0.78 (0.57, 1.06) | 1.90 (1.10, 3.30)* | 1.06 (0.52, 2.16) | 3.04 (1.44, 6.39)** | 2.49 (1.03, 5.98)* |
| High school graduate/GED | 0.94 (0.76, 1.16) | 0.97 (0.75, 1.27) | 1.46 (0.89, 2.40) | 1.21 (0.65, 2.26) | 2.09 (1.02, 4.28)* | 0.96 (0.39, 2.37) |
| Some college (no diploma)/associate degree | 1.15 (0.93, 1.43) | 1.19 (0.90, 1.56) | 1.43 (0.96, 2.14) | 1.61 (0.85, 3.04) | 1.39 (0.84, 2.32) | 1.24 (0.65, 2.34) |
| Undergraduate/graduate degree | 1.00 | 1.00 | 1.00 | 1.00 | 1.00 | 1.00 |
| **Poverty status^h^** |  |  |  |  |  |  |
| <100% (Poor) | 0.93 (0.77, 1.13) | 0.97 (0.77, 1.21) | 0.94 (0.59, 1.52) | 1.09 (0.66, 1.79) | 0.69 (0.40, 1.19) | 0.57 (0.27, 1.23) |
| 100-199% (Near poor) | 0.97 (0.82, 1.15) | 1.01 (0.84, 1.22) | 0.96 (0.62, 1.49) | 1.26 (0.77, 2.07) | 0.99 (0.54, 1.79) | 0.94 (0.45, 1.98) |
| ≥200% (Not poor) | 1.00 | 1.00 | 1.00 | 1.00 | 1.00 | 1.00 |
| Unspecified | 0.93 (0.71, 1.23) | 0.97 (0.71, 1.33) | 0.83 (0.47, 1.46) | 1.10 (0.59, 2.05) | 1.30 (0.53, 3.15) | 0.81 (0.36, 1.83) |
| **Cigarette pack price (US dollar)** | 1.10 (0.99, 1.22) | 1.07 (0.95, 1.22) | 1.20 (0.95, 1.50) | 1.16 (0.91, 1.48) | 1.33 (1.01, 1.75)* | 1.23 (0.85, 1.78) |
| **Smokefree bar law coverage × drinking status** | F_(4, 297)_=0.74; *p*=.564 | F_(4, 297)_=0.37; *p*=.828 | F_(4, 282)_=0.65; *p*=.624 | F_(4, 252)_=1.52; *p*=.198 | F_(4, 256)_=1.01; *p*=.405 | F_(4, 206)_=1.81;*p*=.128 |

*Note.* AOR=adjusted odds ratio; CI=confidence interval.

^a^Current smokers smoked at least 100 cigarettes in their lifetime and smoked “every day” or “some days” now.

^b^Daily smokers smoked “every day” now, or if they smoked “some days”, they smoked on >25 days in the past 30 days.

^c^Nondaily smokers smoked “some days” now and smoked ≤25 days in the past 30 days.

^d^Very light daily smokers are daily smokers who smoked ≤5 cigarettes per day.

^e^Very light nondaily smokers are nondaily smokers who smoked ≤3 cigarettes per day.

^f^Infrequent smokers are nondaily smokers who smoked on ≤8 days in the past 30 days.

^g^Lifetime abstainers had fewer than 12 drinks in lifetime; Former drinkers had at least 12 drinks in lifetime, but none in past year; Current light drinkers drank 1-3 drinks per week in past year; Current moderate drinkers drank 4-14 drinks per week for male and 4-7 drinks per week for female; Current heavy drinkers drank >14 drinks per week for male and >7 drinks per week for female.

^h^Poverty status is a ratio of family income to the appropriate poverty threshold (given family size and number of children) defined by the US Census Bureau. “Poor” adults reported a family income below the poverty threshold. “Near poor” adults had a family income of 100-199% of the poverty threshold. “Not poor” adults reported a family income of 200% of the poverty threshold or greater.

^*^*P*<.05; ^**^*P*<.01.
