# Supplementary material for: Relationship of Smokefree Laws and Alcohol Use with Light and Intermittent Smoking and Quit Attempts among US Adults and Alcohol Users
Source: PLoS One. 2015 Oct 7;10(10):e0137023. doi: 10.1371/journal.pone.0137023 (PMC4596828; doi:10.1371/journal.pone.0137023)
Supplement: S6 Table — (DOCX) [file pone.0137023.s006.docx]

**Supporting information**

**Relationship of Smokefree Laws and Alcohol Use with Light and Intermittent Smoking and Quit Attempts among US Adults and Alcohol Users**

Nan Jiang, MariaElena Gonzalez, Pamela M. Ling, Stanton A. Glantz

**S6 Table. Relationship of smokefree law coverage and drinking with smoking quit attempts among current drinking smokers**

| Subpopulation | Current smoker^a^ | Daily smoker^b^ | Nondaily smoker^c^ | Very light daily smoker^d^ | Very light nondaily smoker^e^ | Infrequent smoker^f^ |
| --- | --- | --- | --- | --- | --- | --- |
|  | AOR (95% CI) | AOR (95% CI) | AOR (95% CI) | AOR (95% CI) | AOR (95% CI) | AOR (95% CI) |
| N | 3935 | 3028 | 898 | 471 | 535 | 338 |
| **Smokefree law coverage score** | 0.85 (0.59, 1.23) | 0.88 (0.58, 1.33) | 0.84 (0.40, 1.74) | 1.48 (0.57, 3.85) | 1.13 (0.45, 2.82) | 0.78 (0.20, 2.99) |
| **Binge drinking^g^** |  |  |  |  |  |  |
| No | 1.00 | 1.00 | 1.00 | 1.00 | 1.00 | 1.00 |
| Yes | 0.82 (0.60, 1.12) | 0.81 (0.57, 1.16) | 0.99 (0.51, 1.92) | 1.21 (0.47, 3.15) | 1.32 (0.59, 2.95) | 1.26 (0.41, 3.92) |
| **Age group (years)** |  |  |  |  |  |  |
| 18-20 | 1.40 (0.83, 2.35) | 1.48 (0.81, 2.71) | 0.80 (0.33, 1.93) | 1.00 (0.30, 3.33) | 0.60 (0.20, 1.75) | 0.53 (0.13, 2.18) |
| 21-24 | 1.97 (1.41, 2.73)*** | 2.15 (1.43, 3.22)*** | 1.12 (0.62, 2.04) | 2.20 (1.02, 4.74)* | 1.05 (0.49, 2.23) | 1.12 (0.36, 3.52) |
| 25-44 | 1.32 (1.11, 1.58)** | 1.28 (1.04, 1.58)* | 1.31 (0.86, 2.01) | 2.05 (1.17, 3.58)* | 1.20 (0.70, 2.05) | 0.77 (0.36, 1.62) |
| 45-64 | 1.00 | 1.00 | 1.00 | 1.00 | 1.00 | 1.00 |
| 65 and above | 1.02 (0.72, 1.43) | 0.95 (0.66, 1.37) | 1.18 (0.56, 2.52) | 1.57 (0.60, 4.12) | 0.53 (0.18, 1.55) | 1.08 (0.31, 3.79) |
| **Female** | 1.06 (0.90, 1.25) | 1.01 (0.84, 1.22) | 1.34 (0.96, 1.87) | 1.13 (0.66, 1.93) | 1.93 (1.21, 3.08)** | 1.93 (1.05, 3.55)* |
| **Race/ethnicity** |  |  |  |  |  |  |
| White, non-Hispanic | 1.00 | 1.00 | 1.00 | 1.00 | 1.00 | 1.00 |
| Black, non-Hispanic | 1.45 (1.15, 1.83)** | 1.43 (1.13, 1.81)** | 1.15 (0.62, 2.12) | 0.61 (0.32, 1.14) | 0.82 (0.39, 1.71) | 2.56 (0.92, 7.12) |
| API and others, non-Hispanic | 1.27 (0.85, 1.89) | 1.02 (0.64, 1.62) | 2.28 (0.99, 5.21) | 0.44 (0.19, 1.03) | 1.98 (0.63, 6.19) | 2.45 (0.68, 8.86) |
| Hispanic | 1.53 (1.12, 2.08)** | 1.60 (1.07, 2.39)* | 0.86 (0.54, 1.36) | 0.70 (0.35, 1.41) | 0.96 (0.54, 1.71) | 1.39 (0.68, 2.83) |
| **Education** |  |  |  |  |  |  |
| 0-12 years (no diploma) | 0.75 (0.57, 1.00)* | 0.71 (0.50, 1.02) | 1.85 (0.99, 3.43) | 1.44 (0.59, 3.53) | 2.32 (1.02, 5.27)* | 2.80 (1.03, 7.57)* |
| High school graduate/GED | 0.98 (0.76, 1.26) | 1.01 (0.74, 1.36) | 1.62 (0.95, 2.74) | 1.16 (0.57, 2.37) | 2.01 (0.90, 4.46) | 1.24 (0.48, 3.25) |
| Some college (no diploma)/associate degree | 1.22 (0.96, 1.55) | 1.29 (0.96, 1.72) | 1.42 (0.93, 2.18) | 1.34 (0.68, 2.62) | 1.22 (0.70, 2.12) | 1.21 (0.59, 2.47) |
| Undergraduate/graduate degree | 1.00 | 1.00 | 1.00 | 1.00 | 1.00 | 1.00 |
| **Poverty status^h^** |  |  |  |  |  |  |
| <100% (Poor) | 0.91 (0.73, 1.13) | 0.93 (0.72, 1.20 ) | 0.96 (0.58, 1.59) | 0.83 (0.45, 1.55) | 0.59 (0.31, 1.11) | 0.45 (0.19, 1.10) |
| 100-199% (Near poor) | 0.94 (0.77, 1.16) | 1.01 (0.81, 1.27) | 0.86 (0.52, 1.45) | 1.25 (0.65, 2.41) | 0.93 (0.48, 1.81) | 0.79 (0.35, 1.80) |
| ≥200% (Not poor) | 1.00 | 1.00 | 1.00 | 1.00 | 1.00 | 1.00 |
| Unspecified | 0.97 (0.70, 1.35) | 1.04 (0.72, 1.50) | 0.77 (0.40, 1.49) | 1.06 (0.50, 2.21) | 1.32 (0.45, 3.90) | 0.72 (0.31, 1.68) |
| **Cigarette pack price (US dollar)** | 1.14 (1.03, 1.27)* | 1.11 (0.97, 1.27) | 1.27 (0.99, 1.61) | 1.04 (0.77, 1.41) | 1.37 (1.01, 1.86)* | 1.28 (0.88, 1.87) |
| **Smokefree law coverage × drinking status** | F_(1, 300)_=0.43; *p*=.510 | F_(1, 299)_=0.30;*p*=.583 | F_(1, 273)_=0.04; *p*=.841 | F_(1, 227)_=0.49; *p*=.484 | F_(1, 242)_=0.94; *p*=.332 | F_(1, 190)_=0.00; *p*=.992 |

*Note.* AOR=adjusted odds ratio; CI=confidence interval.

^a^Current smokers smoked at least 100 cigarettes in their lifetime and smoked “every day” or “some days” now.

^b^Daily smokers smoked “every day” now, or if they smoked “some days”, they smoked on >25 days in the past 30 days.

^c^Nondaily smokers smoked “some days” now and smoked on ≤25 days in the past 30 days.

^d^Very light daily smokers are daily smokers who smoked ≤5 cigarettes per day.

^e^Very light nondaily smokers are nondaily smokers who smoked ≤3 cigarettes per day.

^f^Infrequent smokers are nondaily smokers who smoked on ≤8 days in the past 30 days.

^g^Binge drinkers drank ≥5 drinks on at least one day in the past 12 months.

^h^Poverty status is a ratio of family income to the appropriate poverty threshold (given family size and number of children) defined by the US Census Bureau. “Poor” adults reported a family income below the poverty threshold. “Near poor” adults had a family income of 100-199% of the poverty threshold. “Not poor” adults reported a family income of 200% of the poverty threshold or greater.

^*^*P*<.05; ^**^*P*<.01; ^***^*P*<.001.
