# Supplementary material for: Relationship of Smokefree Laws and Alcohol Use with Light and Intermittent Smoking and Quit Attempts among US Adults and Alcohol Users
Source: PLoS One. 2015 Oct 7;10(10):e0137023. doi: 10.1371/journal.pone.0137023 (PMC4596828; doi:10.1371/journal.pone.0137023)
Supplement: S7 Table — (DOCX) [file pone.0137023.s007.docx]

**Supporting information**

**Relationship of Smokefree Laws and Alcohol Use with Light and Intermittent Smoking and Quit Attempts among US Adults and Alcohol Users**

Nan Jiang, MariaElena Gonzalez, Pamela M. Ling, Stanton A. Glantz

**S7 Table. Relationship of smokefree bar law coverage and drinking with smoking quit attempts among drinking smokers**

| Subpopulation | Current smoker^a^ | Daily smoker^b^ | Nondaily smoker^c^ | Very light daily smoker^d^ | Very light nondaily smoker^e^ | Infrequent smoker^f^ |
| --- | --- | --- | --- | --- | --- | --- |
|  | AOR (95% CI) | AOR (95% CI) | AOR (95% CI) | AOR (95% CI) | AOR (95% CI) | AOR (95% CI) |
| N | 3935 | 3028 | 898 | 471 | 535 | 338 |
| **Smokefree bar law coverage score** | 1.04 (0.79, 1.37) | 1.06 (0.77, 1.46) | 1.07 (0.61, 1.88) | 1.35 (0.66, 2.77) | 1.69 (0.82, 3.48) | 0.81 (0.31, 2.13) |
| **Binge drinking^g^** |  |  |  |  |  |  |
| No | 1.00 | 1.00 | 1.00 | 1.00 | 1.00 | 1.00 |
| Yes | 0.90 (0.70, 1.15) | 0.88 (0.68, 1.16) | 1.07 (0.64, 1.82) | 0.97 (0.47, 2.01) | 1.47 (0.77, 2.79) | 1.17 (0.48, 2.84) |
| **Age group (years)** |  |  |  |  |  |  |
| 18-20 | 1.40 (0.83, 2.36) | 1.49 (0.81, 2.72) | 0.80 (0.33, 1.93) | 0.96 (0.29, 3.16) | 0.59 (0.20, 1.78) | 0.53 (0.13, 2.14) |
| 21-24 | 1.96 (1.41, 2.73)*** | 2.15 (1.43, 3.21)*** | 1.13 (0.62, 2.05) | 2.20 (1.02, 4.73)* | 1.04 (0.48, 2.23) | 1.13 (0.36, 3.54) |
| 25-44 | 1.32 (1.11, 1.58)** | 1.28 (1.04, 1.58)* | 1.32 (0.86, 2.03) | 2.01 (1.15, 3.53)* | 1.20 (0.70, 2.05) | 0.77 (0.36, 1.63) |
| 45-64 | 1.00 | 1.00 | 1.00 | 1.00 | 1.00 | 1.00 |
| 65 and above | 1.02 (0.72, 1.43) | 0.95 (0.66, 1.37) | 1.20 (0.56, 2.54) | 1.61 (0.62, 4.23) | 0.54 (0.18, 1.59) | 1.10 (0.32, 3.79) |
| **Female** | 1.06 (0.90, 1.25) | 1.01 (0.84, 1.22) | 1.32 (0.94, 1.84) | 1.12 (0.65, 1.91) | 1.94 (1.21, 3.09)** | 1.91 (1.05, 3.50)* |
| **Race/ethnicity** |  |  |  |  |  |  |
| White, non-Hispanic | 1.00 | 1.00 | 1.00 | 1.00 | 1.00 | 1.00 |
| Black, non-Hispanic | 1.45 (1.15, 1.83)** | 1.43 (1.13, 1.82)** | 1.12 (0.61, 2.07) | 0.62 (0.33, 1.17) | 0.78 (0.38, 1.61) | 2.57 (0.94, 7.02) |
| API and others, non-Hispanic | 1.25 (0.84, 1.86) | 1.00 (0.64, 1.59) | 2.30 (1.00, 5.27)* | 0.43 (0.18, 1.00) | 2.02 (0.65, 6.27) | 2.43 (0.67, 8.74) |
| Hispanic | 1.52 (1.12, 2.07)** | 1.59 (1.07, 2.36)* | 0.85 (0.53, 1.36) | 0.69 (0.34, 1.38) | 0.93 (0.51, 1.70) | 1.43 (0.71, 2.89) |
| **Education** |  |  |  |  |  |  |
| 0-12 years (no diploma) | 0.75 (0.57, 1.00)* | 0.71 (0.50, 1.02) | 1.86 (1.01, 3.45)* | 1.39 (0.57, 3.37) | 2.40 (1.04, 5.53)* | 2.74 (1.01, 7.45)* |
| High school graduate/GED | 0.98 (0.77, 1.26) | 1.01 (0.75, 1.37) | 1.62 (0.95, 2.74) | 1.13 (0.55, 2.32) | 2.04 (0.91, 4.60) | 1.20 (0.46, 3.13) |
| Some college (no diploma)/associate degree | 1.22 (0.96, 1.54) | 1.28 (0.96, 1.71) | 1.43 (0.93, 2.19) | 1.29 (0.65, 2.56) | 1.21 (0.70, 2.11) | 1.20 (0.59, 2.44) |
| Undergraduate/graduate degree | 1.00 | 1.00 | 1.00 | 1.00 | 1.00 | 1.00 |
| **Poverty status^h^** |  |  |  |  |  |  |
| <100% (Poor) | 0.91 (0.74, 1.13) | 0.94 (0.73, 1.20) | 0.96 (0.58, 1.59) | 0.85 (0.46, 1.56) | 0.58 (0.30, 1.09) | 0.46 (0.19, 1.12) |
| 100-199% (Near poor) | 0.95 (0.77, 1.16) | 1.02 (0.82, 1.27) | 0.88 (0.52, 1.46) | 1.27 (0.66, 2.44) | 0.96 (0.50, 1.87) | 0.78 (0.35, 1.78) |
| ≥200% (Not poor) | 1.00 | 1.00 | 1.00 | 1.00 | 1.00 | 1.00 |
| Unspecified | 0.97 (0.70, 1.34) | 1.04 (0.72, 1.49) | 0.76 (0.39, 1.48) | 1.08 (0.51, 2.29) | 1.23 (0.42, 3.62) | 0.72 (0.31, 1.72) |
| **Cigarette pack price (US dollar)** | 1.12 (1.00, 1.25)* | 1.08 (0.94, 1.24) | 1.25 (0.98, 1.59) | 1.01 (0.74, 1.37) | 1.31 (0.97, 1.77) | 1.27 (0.87, 1.86) |
| **Smokefree bar law coverage × drinking status** | F_(1, 300)_=0.00; *p*=.975 | F_(1, 299)_=0.00; *p*=.981 | F_(1, 273)_=0.65; *p*=.422 | F_(1, 227)_=0.02; *p*=.892 | F_(1, 242)_=4.02; *p*=.046 | F_(1, 190)_=0.06; *p*=.804 |

*Note.* AOR=adjusted odds ratio; CI=confidence interval.

^a^Current smokers smoked at least 100 cigarettes in their lifetime and smoked “every day” or “some days” now.

^b^Daily smokers smoked “every day” now, or if they smoked “some days”, they smoked on >25 days in the past 30 days.

^c^Nondaily smokers smoked “some days” now and smoked on ≤25 days in the past 30 days.

^d^Very light daily smokers are daily smokers who smoked ≤5 cigarettes per day.

^e^Very light nondaily smokers are nondaily smokers who smoked ≤3 cigarettes per day.

^f^Infrequent smokers are nondaily smokers who smoked on ≤8 days in the past 30 days.

^g^Binge drinkers drank ≥5 drinks on at least one day in the past 12 months.

^h^Poverty status is a ratio of family income to the appropriate poverty threshold (given family size and number of children) defined by the US Census Bureau. “Poor” adults reported a family income below the poverty threshold. “Near poor” adults had a family income of 100-199% of the poverty threshold. “Not poor” adults reported a family income of 200% of the poverty threshold or greater.

^*^*P*<.05; ^**^*P*<.01; ^***^*P*<.001.
